# MDNN-DTA: a multimodal deep neural network for drug-target affinity prediction

**DOI:** 10.3389/fgene.2025.1527300

**Published:** 2025-03-20

**Authors:** Xu Gao, Mengfan Yan, Chengwei Zhang, Gang Wu, Jiandong Shang, Congxiang Zhang, Kecheng Yang

**Affiliations:** ^1^ School of Computer and Artificial Intelligence, Zhengzhou University, Zhengzhou, China; ^2^ National Supercomputing Center in Zhengzhou, Zhengzhou, China

**Keywords:** drug-target affinity prediction, multimodal network, attention mechanism, deep learning, feature extraction

## Abstract

Determining drug-target affinity (DTA) is a pivotal step in drug discovery, where *in silico* methods can significantly improve efficiency and reduce costs. Artificial intelligence (AI), especially deep learning models, can automatically extract high-dimensional features from the biological sequences of drug molecules and target proteins. This technology demonstrates lower complexity in DTA prediction compared to traditional experimental methods, particularly when handling large-scale data. In this study, we introduce a multimodal deep neural network model for DTA prediction, referred to as MDNN-DTA. This model employs Graph Convolutional Networks (GCN) and Convolutional Neural Networks (CNN) to extract features from the drug and protein sequences, respectively. One notable strength of our method is its ability to accurately predict DTA directly from the sequences of the target proteins, obviating the need for protein 3D structures, which are frequently unavailable in drug discovery. To comprehensively extract features from the protein sequence, we leverage an ESM pre-trained model for extracting biochemical features and design a specific Protein Feature Extraction (PFE) block for capturing both global and local features of the protein sequence. Furthermore, a Protein Feature Fusion (PFF) Block is engineered to augment the integration of multi-scale protein features derived from the abovementioned techniques. We then compare MDNN-DTA with other models on the same dataset, conducting a series of ablation experiments to assess the performance and efficacy of each component. The results highlight the advantages and effectiveness of the MDNN-DTA method.

## 1 Introduction

Drug discovery is a long-term and complex process (Xue et al., 2018) that often involves the discovery of just one effective molecule among $10^{60}$ potential compounds. Although the wet laboratory method is highly accurate, it requires a considerable amount of time and incurs high costs (Shukla and Tripathi, 2021; Bano et al., 2023; Zhou et al., 2016). In contrast, *in silico* methods are widely recognized for their ability to significantly enhance efficiency and reduce costs (Zhu F. et al., 2024). With the advancement of machine learning theories and the increase in pharmacological data (Pang et al., 2021), numerous effective methods have propelled progress in drug discovery. Among these, Drug-Target Affinity (DTA) prediction stands out as a particularly notable approach (Thafar et al., 2019; Sun et al., 2022; Zhu Z. et al., 2024).

The aim of DTA prediction is to anticipate the interaction strength or binding capacity between drugs and target proteins through computational models or algorithms, also known as binding affinity prediction. This endeavor contributes to understanding the mechanism of drug action, refining drug design, and expediting the drug discovery process (Jarada et al., 2020). Binding affinity is closely related to dissociation constant $\left(K_{d}\right)$ , inhibition constant $\left(K_{i}\right)$ or semi-maximum inhibition concentration $\left(IC_{50}\right)$ (He et al., 2017). The lower the values of these indexes, the stronger the affinity between drugs and target proteins (Cer et al., 2009). Consequently, many researchers utilize the negative logarithm of these metrics ( $pK_{d}$ and $pK_{i}$ ) to quantify the binding affinity (Hua et al., 2023).

Early DTA prediction works primarily rely on the drug structures and the sequence information of the target proteins, typically employing calculation methods (Mousavian and Masoudi-Nejad, 2014; Chen et al., 2016) based on physical and chemical principles to predict their interactions. However, these approaches exhibit high computational complexity and struggle with efficient screening and predicting large-scale compound and protein libraries. During the rapid development of machine learning technology, approaches for predicting DTA based on feature engineering have emerged. Traditional machine learning methods, such as Feed-forward Neural Network (FNN), Support Vector Machine (SVM), and Random Forest (RF) (Lenselink et al., 2017; Mayr et al., 2018; Olayan et al., 2018; He et al., 2017; Chu et al., 2021; Ezzat et al., 2017; Pahikkala et al., 2015), extract features from the structures or sequences of drug molecules and target proteins to establish prediction models. Although traditional machine learning methods are capable of handling high-dimensional and complex data, feature engineering is time-consuming, and the prediction results often lack accuracy. These limitations have hindered the widespread adoption of machine learning in the field of DTA prediction.

With the success of deep learning, various deep learning networks have been employed in DTA prediction. DeepDTA (Öztürk et al., 2018) is initially proposed to extract molecular features from drug molecules using convolutional neural networks (CNNs) and construct DTA prediction models using protein sequence data. Additionally, AttentionDTA (Zhao et al., 2019) similarly employs one-dimensional convolution to capture the underlying relationships between the atoms of drug molecules and the amino acid sequences of proteins, achieving more accurate prediction results compared to DeepDTA. Subsequently, Transformer-based models have stood out in various natural language processing (NLP) tasks. For instance, MT-DTI (Shin et al., 2019) utilizes Transformer to encode word embeddings of drug and protein sequences, aiming to learn hidden associations between the two from raw sequence data. In order to better simulate the biological characteristics of drug molecules and target proteins, GraphDTA (Nguyen et al., 2021) represents compounds as topological graphs and evaluates several types of graph neural network (GNN) variants, including graph convolution network (GCN), graph isomorphism network (GIN) and graph attention network (GAT). DGraphDTA (Sun et al., 2010) extends GraphDTA by representing drug molecules and protein sequences as two-dimensional graphs, incorporating a protein structure prediction method called contact maps to enhance protein structure estimation. Another graph neural network approach, MGraphDTA (Yang et al., 2022), applies multiscale techniques to learn features across various scales, from shallow to deep. By aggregating these features, it generates comprehensive multiscale representations for both drug molecules and proteins, which are then rationalized from a chemical perspective. MDCT-DTA (Zhu Z. et al., 2024) introduces a multi-scale graph diffusion convolution module to capture the complex interactions between drug molecule graph nodes, while a CNN-Transformer block is used to capture the interactions and interdependencies between different amino acids. These graph-based methods not only effectively address the issues of limited complex samples and high computational costs but also overcome the limitations of SMILES (Simplified Molecular Input Line Entry System) in drug representation. Furthermore, molecular graph representation is more aligned with the natural structural description of compounds, which is why GNNs are widely adopted in current mainstream methods.

**FIGURE 1:**
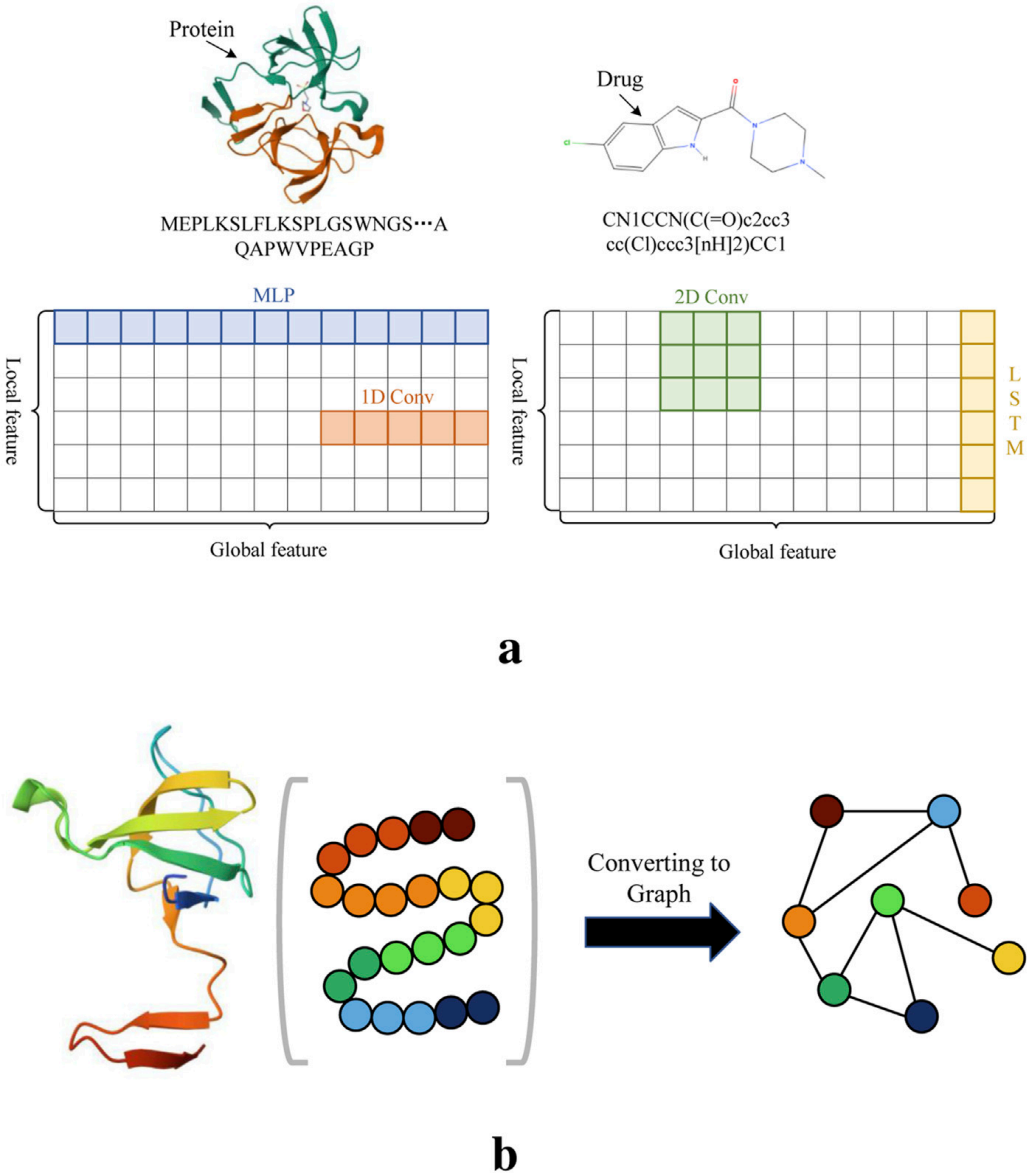
Illustration of several feature extraction methods and amino acid sequences. (A) The receptive fields of several sequence feature extraction methods. (B) The order of amino acids after converting the protein into a graph structure differs from the original structure.

Although all these methods show promising prediction results, they may still face certain challenges. Mainstream biological sequence feature extraction methods include MLP, CNN, LSTM (Chen et al., 2020), each have limitations when applied individually. As illustrated in Figure 1A, relying solely on one of these methods may not be sufficient to fully capture the features of drug molecules and proteins. In particular, both 1D and 2D convolutions typically rely on fixed-size kernels, which limits their ability to effectively extract comprehensive features. While the small kernels can capture local patterns in biological sequences, they overlook global contextual information. 2D convolutions, by stacking layers, can expand the receptive field to capture global information. Nevertheless, longer biological sequences result in significantly higher computational costs. LSTM, with its gating mechanism, effectively manages the transmission and forgetting of information, allowing it to retain local features or short-term dependencies in sequences. While it is possible to modify LSTM to capture longer-range dependencies through design adjustments, its ability to handle long-term dependencies remains limited. In contrast, the fully connected structure of MLP means that all features of the input biological sequence participate in the computation together, allowing for the calculation of global features but neglecting local features. Moreover, there is currently no widely recognized and effective method for converting protein sequences into graph structures that preserves both their global and local patterns. A commonly used graph conversion method represents protein sequences in a graph structure, where nodes denote amino acids and edges represent interactions between them. However, the sequence information of proteins is linear, and the order of amino acids is crucial. GCNs are primarily designed for graph-structured data, focusing on the topological relationships between nodes rather than directly modeling the sequential information of the nodes. As shown in Figure 1B, when converting the amino acids of a protein sequence into nodes of a graph structure, there is a significant difference from the original order of the amino acids. In contrast, drug molecules can naturally be represented using graph structures. Most DTA prediction methods commonly convert their sequences into graph structures using bioinformatics tools (Jiang et al., 2020; Zhang et al., 2022; Zhu et al., 2023b; Wang et al., 2024). This method is inherently tied to the biochemical properties of the molecules. Drug molecules can be perceived as structures comprising atoms and chemical bonds between them. Graph structures adeptly capture this interconnection between atoms and bonds, with nodes symbolizing atoms and edges representing chemical bonds. Given these advantages, we employ this approach in our study to process drug sequences.

To address the aforementioned issues, we decide to choose CNNs to identify important patterns and substructures in the sequences through convolutional and pooling layers, thereby extracting local features from protein sequences. Utilizing a CNN-based approach as a local feature extractor for proteins aids in preventing potential information loss and unreasonable structural representations that can occur during the conversion of protein sequences into graph formats. Furthermore, we design a global feature extractor that utilizes an MLP, which works in conjunction with the previously mentioned local feature extractor to form a Protein Feature Extraction (PFE) Block, allowing for the acquisition of more comprehensive information from protein sequences.

The main contributions of this study can be summarized as follows:

• A multimodal neural network-based MDNN-DTA prediction model is proposed based on the different biochemical properties of drug molecules and target proteins. This model utilizes GCN and CNN methods to handle the feature representations of drug and protein sequences, enabling efficient prediction of DTA;

• We presents a Protein Feature Extraction (PFE) block to delve into the global and local features of the target protein’s linear sequence. Additionally, we introduce a pre-trained model to supplement the attribute information of the sequence at the molecular level to obtain more detailed, robust, and multidimensional representation of protein features;

• By incorporating a protein feature fusion (PFF) block based on an attention mechanism, MDNN-DTA can efficiently integrate multi-scale features of protein sequences.

## 2 The proposed method

### 2.1 Model architecture

**FIGURE 2:**
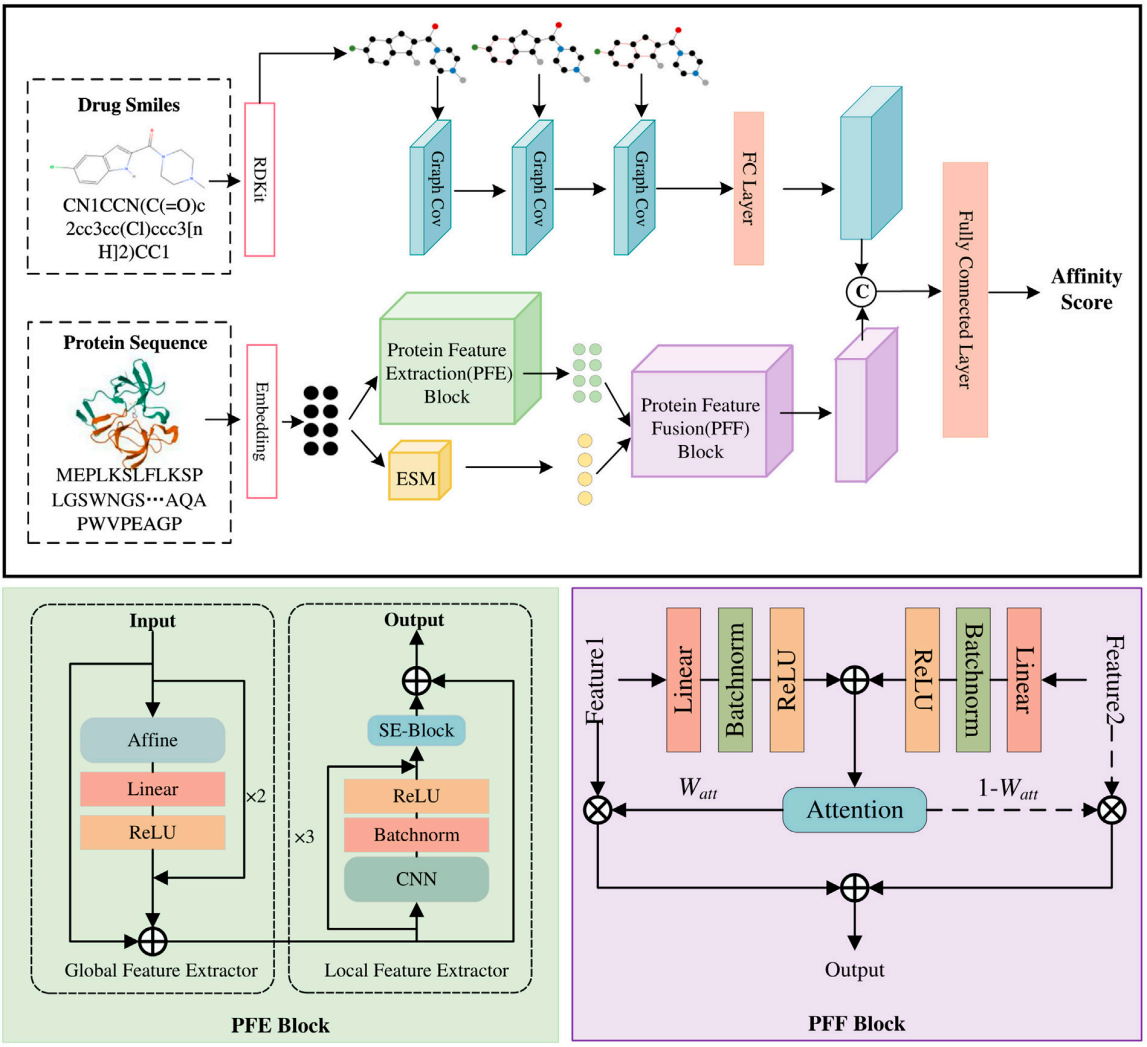
The model architecture of MDNN-DTA. The MDNN-DTA includes a Drug Feature Extraction block as well as a Protein Feature Extraction (PEE) Block and a Protein Feature Fusion (PEF) Block designed by us.

Our MDNN-DTA model consists of three parts: sequence input, feature extraction, and affinity prediction (Figure 2). The feature extraction process is the focus of our work, encompassing the Drug Feature Extraction Block along with the PFE block and PEE block for processing protein sequences. In MDNN-DTA, drug molecules are represented in SMILES format. The drug branch converts each molecule’s sequence into a graph structure and utilizes a GCN-based Drug Feature Extraction Block to capture its sequence features. Meanwhile, proteins are processed by embedding their amino acid sequences, and the proposed PFE Block extracts both their global and local features. Furthermore, the study integrates a pre-trained protein model, ESM, to augment the feature extraction process of protein sequences. MDNN-DTA enhances protein feature representation by incorporating multi-scale protein features through an attention-based Protein Feature Fusion PFF Block. Lastly, the features of drug molecules and target proteins are merged and fed into multiple fully connected layers for affinity prediction. Detailed descriptions of each component in the model will be provided in the following sections.

### 2.2 Input representation

#### 2.2.1 Drug representation

**FIGURE 3:**
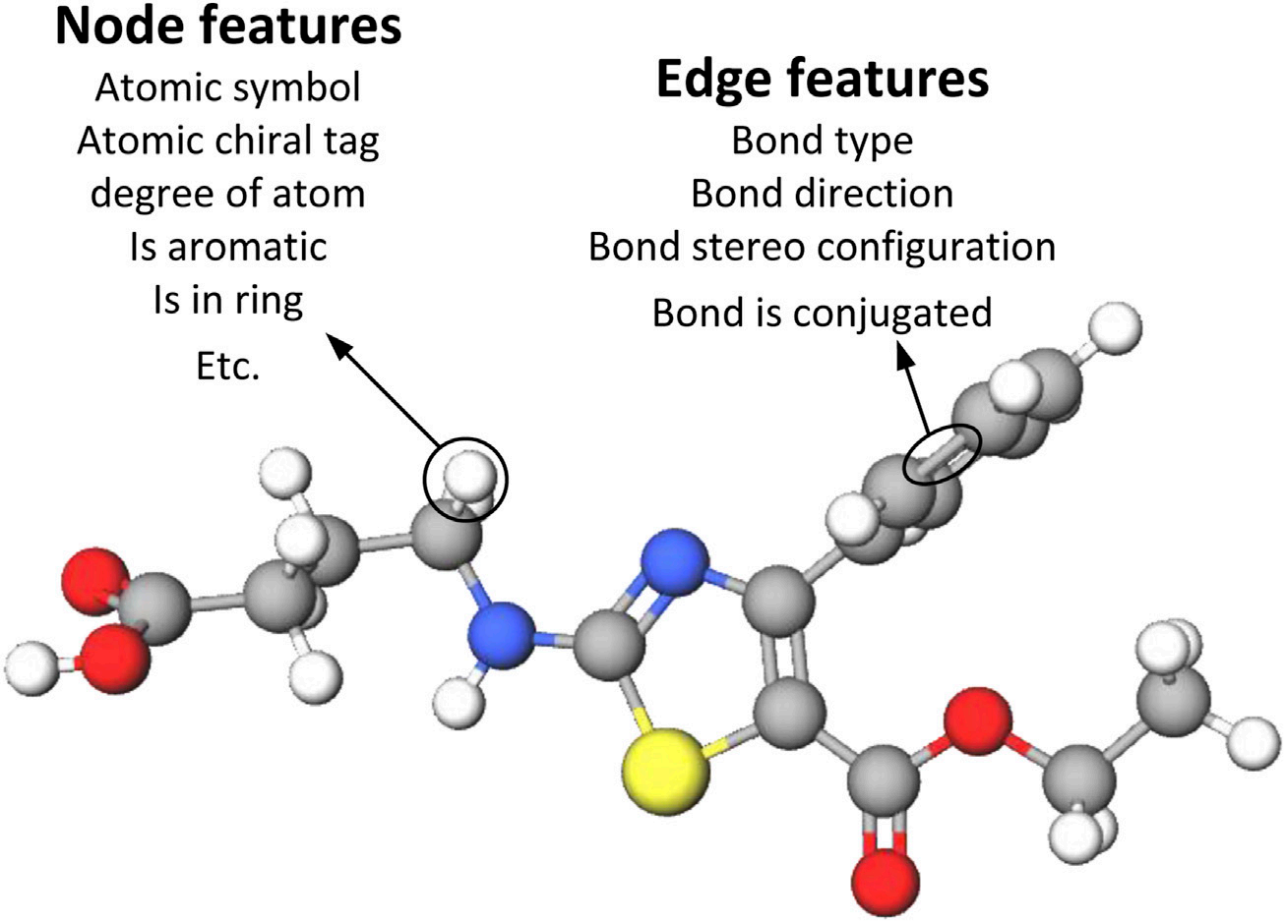
Representation of drugs using node and edge features. Atomic level drug molecular graph with 9 node features and 4 edge features.

The input drug molecules are in SMILES format, a specification that succinctly describes molecular structures using ASCII strings. We employ TorchDrug (Zhu et al., 2022) and RDKit libraries to extract nodes, edge features, and adjacency matrices from the SMILES of drug molecules. Figure 3 illustrates the methodology employed in this paper to construct the node and edge features of the drug graphs. The node and edge features of drug molecules are represented using one-hot vectors, encompassing nine types of node features and four types of edge features, as detailed in Table 1. These features have been utilized in prior studies (Wu et al., 2021; Wu et al., 2024; Suviriyapaisal and Wichadakul, 2023).

**TABLE 1:** Node and edge one-hot features for drug representation.

Type	Feature	Size
Node features	Atomic chiral tag	4
	degree of atom	8
	Number of formal charge	11
	Number of explicit and implicit Hs	7
	Number of radical electron	8
	Atom hybridization	8
	Is aromatic	1
	Is in ring	1
Edge features	Bond type	4
	Bond direction	7
	Bond stereo configuration	6
	Bond is conjugated	1

#### 2.2.2 Protein representation

For protein sequences, we construct an integer dictionary of FASTA sequences in order to map each character to an integer. For example, the MLK3 (Rattanasinchai and Gallo, 2016) protein subsequence “VQIARGM” can be encoded as [22, 17, 9, 1, 18, 7, 13] according to the protein dictionary {“V”:22, “Q”:17, “I”:9, “A”:1, “R”:18, “G”:7, “M”:13}. This encoding method enables the representation of the protein sequence as an integer sequence. To facilitate training, we decide that the maximum length of the protein sequence is fixed at 1,200, so that the maximum length covers at least 80 % of the proteins, as suggested by earlier studies (Öztürk et al., 2018). Each integer is subsequently mapped to a learnable 128-dimensional vector through an embedding layer.

Since protein structures are much more complex compared to drug molecules, this study leverages a pre-trained model called Evolutionary Scale Modeling (ESM) (Meier et al., 2021) to extract more precise and comprehensive features from protein sequences. ESM is a Transformer-based protein language model that excels in capturing detailed feature representations from sequences, surpassing traditional amino acid feature extraction methods (Suviriyapaisal and Wichadakul, 2023). Details about ESM will be discussed in the subsequent sections.

### 2.3 Drug feature extraction block

Drug molecules are non-Euclidean chemical structures composed of entities (atoms) and relationships (bonds), containing rich semantic information and intricate spatial structures. In this paper, we construct a Drug Feature Extraction Block based on GCN. We define the drug molecule graph as G=(V,E) , where V is the set of vertices and E is the set of edges. We adhere to the common approach by representing drug atoms as V , with chemical bonds between atoms serving as E . The traditional GCN method (Wu et al., 2022) is the result of an improvement in GNN, mainly by applying convolutional operations to the molecular graph structure. Its propagation rule is illustrated in Equation 1.

$$H^{\left(l+1\right)}=\sigma \left({\tilde{D}}^{-\frac{1}{2}}\tilde{A}{\tilde{D}}^{-\frac{1}{2}}H^{\left(l\right)}W^{\left(l\right)}\right)$$ (1)

$$\tilde{A}=A+I$$ (2)

where $\tilde{A}$ is the adjacency matrix of the graph, defined as Equation 2. This implies that the feature extraction process on the graph’s nodes necessitates combining both the information from neighboring nodes (denoted by A ) with the node’s own features (denoted by I ). This method ensures that each node’s intrinsic properties are considered alongside the information from its neighboring nodes during the feature extraction process. The $\tilde{D}$ is a diagonal matrix where each element on the diagonal represents the corresponding degree of a node in the graph. The $H^{\left(l\right)}$ denotes the feature output of a node in layer l . The $W^{\left(l\right)}$ corresponds to a linear fully connected layer and is a learnable weight parameter. The σ is a ReLU activation function.

In order to enrich the feature representation of nodes and edges in the drug graph, the study incorporates the edge features of drug molecules into the graph message-passing process of GCN with Equation 3.

$$H^{\left(l+1\right)}=\sigma \left({\tilde{D}}^{-\frac{1}{2}}\tilde{A}{\tilde{D}}^{-\frac{1}{2}}\left(H^{\left(l\right)}+EW_{e}^{\left(l\right)}\right)W^{\left(l\right)}\right)$$ (3)

where the $H^{\left(l\right)}$ + $EW_{e}^{\left(l\right)}$ replaces the $H^{\left(l\right)}$ in Equation 1. The E in the replacement part denotes the edge features of the drug graph and $W_{e}^{\left(l\right)}$ is a learnable weight parameter. The edge features of drug molecules comprise information such as chemical bond types, bonding directions, stereo configurations (Kim, 2021), etc. Integrating these features into the GCN method can better simulate the chemical properties of drug molecules and enhance the model’s ability to model drug properties.

### 2.4 Protein feature extraction block

Sequence features of proteins include global and local features. Its local features refer to some local patterns in the protein sequence, such as structural domains, helical domains, and so on. CNNs can construct textual features by incorporating spatial correlations between the features, thereby effectively capturing these localized features in protein sequences. Therefore, the PFE Block adopts a CNN-based approach to extract features from protein sequences. However, traditional CNN methods are limited by their convolutional kernels and can only extract local features of the sequence, ignoring the global features of the proteins, such as the protein’s folding state and functional domains along the entire chain. To address these limitations, the proposed PFE Block employs two sub-modules: a global feature extractor and a local feature extractor, as shown in Figure 2.

#### 2.4.1 Global feature extractor

The global feature extractor consists of an affine block, a L-R layer (a linear layer and a ReLU activation layer), and the residual method. Inspired by ResMLP (Touvron et al., 2022), we utilize residual connections to enhance the learning capability of the multi-layer perceptron (MLP) architecture. The global feature extractor aims to extract the global features of the entire protein amino acid sequence through the MLP without disrupting the sequence order, and then pass these features to subsequent operations. The architecture can be expressed as Equation 4, where $X_{in}$ and $X_{\mathrm{out}}\;\in R^{L\times C}$ are the inputs and outputs of the extractor, respectively. L is the length of the protein feature vector, and C is the size of the embedding channel.

$$X_{\mathrm{out}}=X_{in}+{\left[FC_{1}\left(AF\left(X_{in}\right)\right)\right]}_{\left(2\right)}$$ (4)

$$AF\left(X\right)=Diag\left(\alpha \right)X+\beta$$ (5)

As illustrated in Equation 4, AF denotes an Affine-block, which is used to perform a linear transformation of the input features. AF is defined as Equation 5, where diag creates a diagonal matrix and both α and β are trainable weighting vectors. $FC_{1}\left(\cdot \right)$ comprises a linear layer and a ReLU layer and ${\left[\cdot \right]}_{\left(2\right)}$ means that the part of [⋅] loops twice. The main reason why Equation 4 can capture the global features of a protein sequence is due to its fully linked character. The input feature vector $X_{in}$ is subjected to a linear transformation through an affine block first and then passed to the L-R layer, which linearly combines all the input features to capture the global relationships of the sequence. Finally, we add the original input $X_{in}$ to the output of the L-R layer using the residual method, ensuring that the original information is preserved from layer to layer.

#### 2.4.2 Local feature extractor

Our local feature extractor combines the advantages of CNN methods to capture local pattern features of proteins through appropriately sized convolutional receptive fields. The application of an SE attention mechanism enables this extractor to focus on the importance of various channels, thereby generating feature vectors that are more aligned with the natural amino acid sequence. The local feature extractor consists of three CNN Blocks and a SE-Block (Hu et al., 2018), and its extraction process is represented as Equation 6.

$$X_{\mathrm{CNN}}=X_{\mathrm{out}}+Att_{se}\left({CNN}_{\left(3\right)}\left(X_{\mathrm{out}}\right)\right)$$ (6)

where $X_{\mathrm{CNN}}\in R^{L\times C}$ is the output of the local feature extractor, L is the length of the protein feature vector, and C is the size of the embedding channel. The CNN contains three CNN Blocks, each consisting of a 1D convolutional layer, a batch normalization layer, and a ReLU layer. The $X_{\mathrm{out}}\in R^{L\times C}$ is the output of the global feature extractor, and $Att_{se}$ represents a SE-Block that dynamically learns the importance of each channel through two main steps: squeezing and excitation. Its implementation is shown in Equation 7.

$$z_{c}=F_{sq}\left(u_{c}\right)=\frac{1}{H\times W}\overset{H}{\underset{i=1}{\sum }}\overset{W}{\underset{j=1}{\sum }}u_{c}$$ (7)

$$s=F_{ex}\left(z,W\right)=\sigma \left(W_{2}\delta \left(W_{1}z\right)\right)$$ (8)

where the squeeze operation is the compression of a H×W×C feature vector $u_{c}$ into a 1×1×C feature vector z by global average pooling, as defined by function $F_{sq}\left(\cdot \right)$ in Equation 7. The symbol $u_{c}$ denotes the c -th feature map of the input feature U , which is the output of ${CNN}_{\left(3\right)}\left(X_{\mathrm{out}}\right)$ , and $z_{c}$ is the c -th element of z . The excitation process is computed as shown in Equation 8, where δ is the ReLU activation function, σ is the sigmoid activation function, and $W_{1}$ and $W_{2}$ are two learnable weight matrices. The excitation operation processes the vector z obtained in the previous step through two fully connected layers to learn the nonlinear interactions between the channels, filtering the non-dominant features and obtaining the channel weight s . Specifically, the first fully connected layer compresses the C channels into C/r channels to reduce computational load, followed by a ReLU activation layer. The second fully connected layer restores the channel count back to C , and the sigmoid activation function is then applied to obtain the weight s . The final output s has dimensions 1×1×C , representing the weights assigned to each of the C feature maps within the feature map U . The parameter r , such as 16, indicates the compression ratio.

**FIGURE 4:**
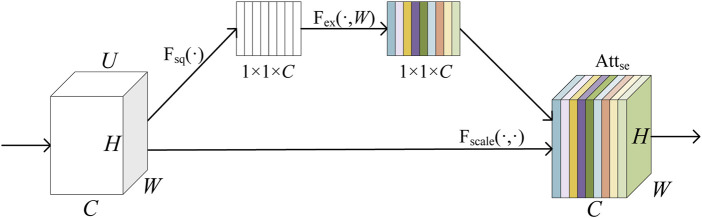
Schematic diagram of SE-Block. $F_{sq}\left(\cdot \right)$ represents the process of squeeze and $F_{ex}\left(\cdot ,W\right)$ represents the process of excitation.

The flow of the SE-Block is illustrated in Figure 4. Here, $F_{sq}\left(\cdot \right)$ represents the process of multiplying each feature map in U by its corresponding weight s , resulting in the final output of the SE-Block, denoted as $Att_{se}$ .

Specifically, the local feature extractor utilizes a convolutional layer to capture the local patterns of the protein sequence and then adjusts the channel weights adaptively through the SE attention mechanism, which enhances the importance of the local features. Additionally, to ensure the integrity of the features, the local feature extractor also uses the residual method to add the global information of the protein sequence directly to the local features.

#### 2.4.3 ESM pre-trained protein language model

Although protein features can be extracted independently based on the nature of each amino acid, such as residue signatures, aliphatic group, and polarity, the function of a protein changes when amino acids are linked together to form a complex structure. Hence, the study employs a pre-trained model known as ESM (Meier et al., 2021; Lin et al., 2023), which is a deep neural network model specialized for processing protein sequences. Biologically, the protein sequence of an organism is not a random arrangement of amino acids. Instead, it is shaped and constrained by the principles of natural selection. Compared with the amino acid embedding approach, the ESM protein language model incorporates biological knowledge such as chemical properties, structural domains, and conserved sites of amino acids. This enhancement improves the model’s capacity to comprehend and represent protein sequences.

Specifically, the ESM-1v utilized in this paper is a generalized protein language model trained on the UR90 dataset (Medrano and Urton, 2018) with 250 million protein sequences. This dataset comprises 98 million distinct protein sequences, with each residue containing 1,280 dimensions of embedded features. However, ESM has a limitation on sequence length because it is trained with a fixed context size of 1,024 tokens for positional embedding. Therefore, for protein sequences longer than 1,024 amino acids, a random sampling method will be employed to truncate the protein sequence length to 1,024 tokens per training batch in order to obtain sample sequences. We refer to the features extracted by the ESM-1v pre-trained model as complementary features of the protein sequence.

### 2.5 Protein feature fusion block

**FIGURE 5:**
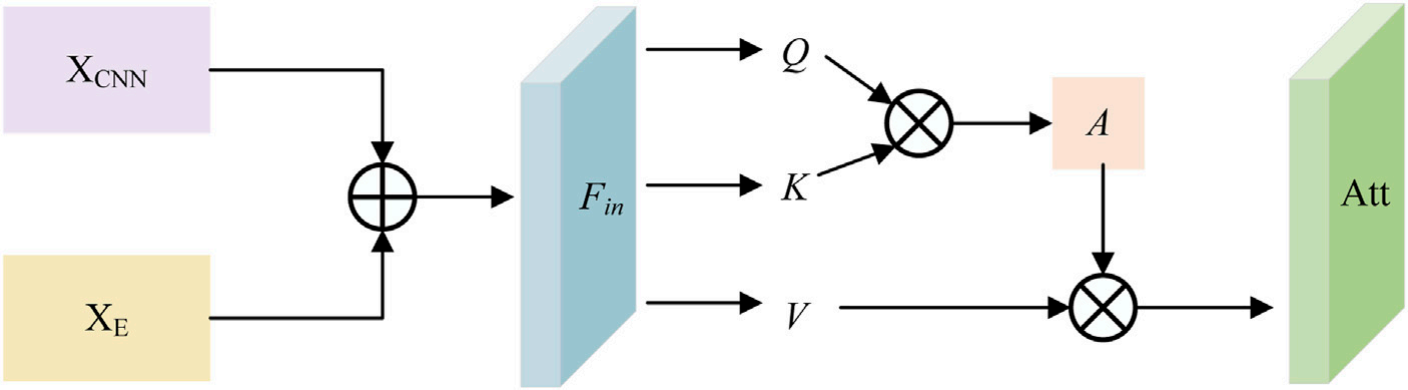
Schematic diagram of Attention Block. $F_{in}$ represents the result of summing the two features, while A denotes an attention matrix.

From the previous two sections, we can see that our model captures two parts of the protein sequence features: the sequence features extracted by the PFE Block, which include both global and local features, and the biological characteristics supplemented by the ESM pre-trained model. To integrate these two parts of the features into a more complete protein representation and enhance the representational power of the model, we construct a PFF block based on attention, as demonstrated in Figure 2. The specific representation can be formulated as Equations 9, 10.

$$X_{f}=X_{\mathrm{CNN}}\times W_{\mathrm{att}}+X_{e}\times \left(1-W_{\mathrm{att}}\right)$$ (9)

$$W_{\mathrm{att}}=Att\left(FC_{2}\left(X_{\mathrm{CNN}}\right)+FC_{2}\left(X_{E}\right)\right)$$ (10)

where $X_{f}$ is the final feature vector of the protein sequence which represents the output of the PFF Block. Given $X_{\mathrm{CNN}}\in R^{L\times C}$ as the output of the PFE Block and $X_{E}\in R^{L\times C}$ as the complementary features extracted by the ESM pre-trained model. Consistent with the aforementioned discussion, they both have the same dimension L×C . The $W_{\mathrm{att}}$ is a weight matrix, and Equation 10 gives the formula for it, where $FC_{2}\left(\cdot \right)$ consists of a linear layer, a ReLU layer, and a batch normalization layer. Att is a self-attention mechanism used to calculate attention weights and the process is given as Equation 11.

$$Att=softmax\left(\frac{QK^{T}}{\sqrt{d_{k}}}\right)V$$ (11)

where Q , K , and V have the same values and are obtained by performing a sum operation on $X_{\mathrm{CNN}}$ and $X_{E}$ . The Q , K , and V are the query matrix, key matrix, and value matrix of the input matrices, respectively. Firstly, the dot product of the query matrix Q and the key matrix K is calculated to obtain an L×L matrix, where each element represents the similarity between two elements in the sequence. Then, it is scaled by dividing by $\sqrt{d_{k}}$ to prevent gradient explosion due to excessively large dot product results, where $d_{k}$ is the dimension of K . Finally, the softmax function is applied, and a weighted sum is performed with V to obtain the attention weights. The computation flow of the attention block is illustrated in Figure 5.

This attention-based PFF Block provides a method for effectively integrating the two parts of the protein sequence features, thereby enabling better interaction between protein features and drug features and improving the accuracy of the affinity prediction model.

### 2.6 Affinity prediction module

After the drug and target protein sequences undergo their respective feature extraction processes, their features need to be fused to predict affinity. In MDNN-DTA, we concatenate the drug and protein features before inputting them into multiple fully connected layers to obtain the affinity score. The model utilizes three fully connected layers with 1,024, 512, and 128 neurons, respectively. Each linear layer is connected by batch normalization layers and ReLU activation functions. Meanwhile, the model training process employs the Mean Square Error (*MSE*) as the loss function, calculated as the sum of squares of the difference between the predicted value (P) and the true value (Y) , as shown in Equation 12.

$$MSE=\frac{1}{n}\overset{n}{\underset{i=1}{\sum }}{\left(P_{i}-Y_{i}\right)}^{2}$$ (12)

## 3 Experimental results

### 3.1 Datasets

In this study, two widely recognized and publicly available datasets, Davis (Davis et al., 2011; Zhu et al., 2023a), KIBA (Öztürk et al., 2018; Tang et al., 2014) and BindingDB (Gilson et al., 2016), are utilized to carry out model performance evaluation experiments. The specific information of the datasets is presented in Table 2.

**TABLE 2:** Statistics of the two datasets.

Datasets	Drugs	Proteins	Interactions
Davis	68	442	30056
KIBA	2,111	229	118254
BindingDB	9,864	1,088	42203

The Davis dataset comprises 442 kinase proteins, 68 inhibitors, and 30,056 drug-target interactions with binding affinities obtained by measuring $K_{d}$ values expressed in nanomoles. In order to describe the relationship between $K_{d}$ and affinity more graphically, $K_{d}$ and $pK_{d}$ are transformed into a logarithmic space in this paper. The larger the value of $pK_{d}$ , the stronger the binding affinity is. The formula for $pK_{d}$ is as shown in Equation 13

$$pK_{d}=-\log_{10}\left(\frac{K_{d}}{10^{9}}\right)$$ (13)

The KIBA dataset also comprises kinase inhibitor interactions, but the difference between the two datasets lies in its construction from various sources of biological activity. The metrics of the KIBA dataset include inhibition constants $K_{i}$ , $K_{d}$ , and half-maximal inhibitory concentrations $IC_{50}$ . The KIBA score is derived by optimizing these values and is employed to determine drug-target binding affinity. The original KIBA dataset is quite extensive, and the filtered dataset was obtained after removing all drugs and proteins with fewer than 10 interactions. The filtered KIBA dataset, utilized in this study, comprises 118,254 drug-target interactions with 2,111 unique drugs and 229 unique proteins. BindingDB is an open bioinformatics database that focuses on the binding affinity between small molecules and protein targets. As Table 2 shown, the BindingDB dataset contains 9,864 compounds, 1,088 target proteins, and 42,203 interaction data points.

To ensure the fairness of the experiment and the reliability of the evaluation, the study uses a five-fold cross-validation (Shar et al., 2016) approach for model comparison and selection. In each fold, all methods share the same training, validation, and test sets. The entire dataset is randomly divided into five parts, with four parts used for five-fold cross-validation and the remaining part serving as an independent test dataset. Subsequently, a candidate model is selected to evaluate its performance on an independent test set after the training is completed. Firstly, the experiment selects the candidate model with the best average results from the validation set results for each fold. Secondly, the candidate models are evaluated using the weights of each fold for predicting the independent test set. Finally, the final results are calculated by averaging the test results obtained for each fold.

### 3.2 Experimental metrics

In this paper, we use *MSE*, Consistency Index (*CI*) and the *Pearson* correlation coefficient as the main metrics for DTA prediction task. The *MSE*, as shown in Equation 12, is a commonly used metric to quantify the difference between the predicted and true values. A smaller *MSE* indicates that the predicted values of the model are closer to the true values, reflecting better model performance. The *CI* value evaluates the predictive capability of the model and is defined as the proportion of labeled pairs for which the predicted result aligns with the actual result. A larger value of *CI* indicates a stronger predictive ability of the model. The formula for *CI* is defined as shown in Equation 14, where $b_{x}$ represents the predicted value of the larger affinity $d_{x}$ , $b_{y}$ represents the predicted value of the smaller affinity $d_{y}$ , z is the normalization constant, and h(x) is the step function as described in Equation 15.

$$CI=\frac{1}{z}\underset{d_{x}>d_{y}}{\sum }h\left(b_{x}-b_{y}\right),$$ (14)

$$h\left(x\right)=\left\{\begin{array}{c} 1\;x>0\\ 0.5\;x=0\\ 0\;x<0 \end{array}\right.$$ (15)

The *Pearson* correlation coefficient, as described in Equation 16, where cov is the covariance between the predicted value p and the true value y , and σ(p)σ(y) denotes the standard deviation of the predicted value p and the true value y respectively. This coefficient measures the strength of the linear relationship between two variables, specifically reflecting the correlation between the predicted and true values in this context.

$$Pearson=\frac{cov\left(p,y\right)}{\sigma \left(p\right)\sigma \left(y\right)}$$ (16)

### 3.3 Experimental settings

All experiments in this study are conducted on an Nvidia RTX 4090 machine. MDNN-DTA is implemented using Python 3.8.18 and PyTorch 1.13.1 and trained using the AdamW optimizer (Loshchilov, 2017). The learning rate is initially set to 0.001 and decays by 80 % every 100 epochs to facilitate learning complex patterns during later training iterations. The weight decay parameter of the two benchmark datasets used for the study is set to 0.01, ensuring regularization during training. All parameters are set as shown in Table 3. The Drug encoder layer refers to the number of GCN layers in the Drug Feature Extraction Block, and we also discuss the rationale for selecting three GCN layers in subsequent experiments. The layer settings for the PFE-global Layer and PFE-local layer are based on the optimal results we adjust during the model development process. The maximum length of the protein and the embedding dimension are borrowed from other good models (Nguyen et al., 2021; Yang et al., 2022; Qian et al., 2023), and the convolutional kernel size of the CNN is set to 7, as explained in the experimental analysis in the following sections. Dropout (D) and Dropout (P) refer to the dropout rates used for the drug and protein feature vectors before fusion. We also analyze their optimal values in the experiments.

**TABLE 3:** Parameterization of our model.

Parameter	Setting
Embedding Size	128
Drug encoder layer	3
PFE-global layer	2
PFE-local layer	3
Feature size in GCNs	[1,024, 512, 128]
Hidden size in PFE-global	[1,024, 512, 128]
Max length of protein	1,200
Protein embedding dim	128
CNN’s kernel size	7
Epoch	1,000
Optimizer	AdamW
Batchsize	128
Learning rate	0.001
Weight decay	0.01
Dropout(D)	0.2
Dropout(P)	0.05

### 3.4 Comparison with the state-of-the-art methods

To assess the superiority of MDNN-DTA, we compared it with some state-of-the-art models, DeepDTA (Öztürk et al., 2018), WideDTA (Öztürk et al., 2019), MT-DTI (Shin et al., 2019), GraphDTA (Nguyen et al., 2021), etc. These models utilize various approaches, such as single networks of CNN, Transformer, GNN, GCN, or a combination of different networks. The evaluation metrics include MSE, CI, and Pearson correlation coefficient. To ensure a fair comparison, we used the same benchmark datasets of Davis, KIBA and BindingDB as well as the same performance metrics for evaluation. The results of our experiments are shown in Table 4, where the parameters are calculated from the model architecture.

**TABLE 4:** Model performance on the Davis, KIBA and BindingDB datasets.

Dateset	Model	Drug	Protein	MSE ↓	CI ↑	Pearson ↑	Params	Time
Davis	DeepDTA (2018)	CN	CN	0.261	0.878	0.821	1.9M	1.95 s
	WideDTA (2019)	CN	CN	0.262	0.886	0.820	2.2M	1.94 s
	MT-DTI (2019)	Trans	CN	0.245	0.887	0.835	-	1.99 s
	GraphDTA (2021)	CN	GI	0.229	0.893	0.848	1.3M	1.95 s
	GEFA (2021)	GC	GC	0.228	0.893	0.847	1.1M	2.00 s
	${\text{S}}^{2}$ GC + SAGE (2022)	GN	GN	0.227	0.895	0.846	-	1.96 s
	SMT-DTA (2022)	Trans	Trans	0.219	0.890	0.857	285.7M	2.02 s
	IEDGEDTA (2023)	GC	GC	0.216	0.897	0.855	7.5M	1.96 s
	TC-DTA (2024)	CN	Trans	0.231	0.885	0.853	43.9M	2.01 s
	MDNN-DTA	GC	CN	**0.209**	**0.901**	**0.860**	6.9M	1.97 s
KIBA	DeepDTA (2018)	CN	CN	0.194	0.863	0.843	1.9M	7.17 s
	WideDTA (2019)	CN	CN	0.179	0.750	0.856	2.2M	7.19 s
	MT-DTI (2019)	Trans	CN	0.152	0.882	0.891	-	7.22 s
	GraphDTA (2021)	CN	GI	0.147	0.882	0.886	1.3M	2.11 s
	SMT-DTA (2022)	Trans	Trans	0.154	**0.894**	0.882	285.7M	7.23 s
	IEDGEDTA (2023)	GC	GC	0.139	0.890	0.892	7.5M	7.20 s
	BiCompDTA (2023)	GC	GC	0.137	0.892	0.892	2.5M	7.17 s
	TC-DTA (2024)	CN	Trans	0.177	0.876	0.854	43.9M	7.19 s
	MDNN-DTA	GC	CN	**0.135**	0.893	**0.896**	6.9M	7.21 s
BindingDB	DeepDTA (2018)	CN	CN	0.685	0.863	-	1.9M	2.74 s
	AttentionDTA (2019)	CN	CN	0.603	0.852	0.839	-	2.71 s
	GraphDTA (2021)	CN	GI	0.557	0.857	0.847	1.3M	2.69 s
	DoubleSG-DTA (2023)	CN	CN	0.533	0.862	0.852	1.4M	2.71 s
	IEDGEDTA (2023)	GC	GC	0.528	0.865	0.859	7.5M	2.74 s
	TC-DTA (2024)	CN	Trans	0.621	0.859	0.837	43.9M	2.77 s
	MDNN-DTA	GC	CN	**0.512**	**0.873**	**0.868**	6.9M	2.75 s

CN represents CNN, GN represents GNN, GC represents GCN, trans represents transformer, and GI represents GIN.Bold values indicate the best results.

Table 4 demonstrates that MDNN-DTA achieves the best results on all benchmark datasets. On the Davis dataset, compared to the IEDGEDTA model, which uses GCN methods to extract features from drug and protein sequences, MDNN-DTA shows an improvement of 3.2 % in MSE, 0.4 % in CI, and 1.1 % in Pearson coefficient. Compared to the SMT-DTA model, which does not rely on graph convolution, the MSE is reduced by 4.5 % , while the CI and Pearson coefficient are improved by 1.2 % and 0.3 % , respectively.

Similarly, on the KIBA dataset, MDNN-DTA also achieves optimal results. The proposed model reduces the MSE by 1.2 % compared to the GCN-based BiCompDTA model, the CI increases by 0.2 % , while the Pearson coefficient rises by 0.4 % . Although MDNN-DTA does not consider the 3D structure of drug molecules and target proteins, but rather extracts features based on their one-dimensional sequences, it still performs comparably to the BiCompDTA model that takes 3D structures into account.

Considering the BindingDB dataset, the MDNN-DTA model reaches an MSE of 0.512, which is 15.1 % lower than the best sequence model, AttentionDTA. Its CI and Pearson coefficient are 0.873 and 0.868, respectively, surpassing those of AttentionDTA by 2.4 % and 3.4 % . Compared to the best GCN-based IEDGEDTA model, the CI improved by 0.9 % and the Pearson coefficient by 0.1 % , while the MSE decreased by 3.0 % . Notably, despite IEDGEDTA utilizing the same pre-trained ESM model, MDNN-DTA surpasses it comprehensively across all evaluation metrics.

Additionally, Table 4 shows the inference time per batch during the model training process. We compare the models using the same batch size on the same dataset. Overall, models using the Transformer architecture have slightly higher inference times per batch compared to those using GCN and CNN methods. The inference time per batch is positively correlated with the number of interactions in the dataset. That is, when there are more interaction relationships in the dataset, the inference time increases accordingly. This result indicates that while the Transformer architecture performs better on complex relationships, it also comes with higher computational overhead.

All the experimental results emphasize the significance of choosing suitable feature extraction methods tailored to drug molecules and target proteins. The linear sequential nature of protein sequences significantly contributes to improving the accuracy of the model’s predictions.

### 3.5 Ablation experiment

For the ablation experiments, we first analyze the effectiveness of both the PFE Block and the PFF Block in MDNN-DTA. Subsequently, we conducted ablation analysis on each component of the PFE Block to enhance our understanding of the protein feature extractor. Lastly, we explore the importance of ESM pre-training models in feature extraction and perform related ablation and comparison experiments. Lastly, concerning the Drug Feature Extraction Block, we also explore the influence of the depth of GCN on the prediction accuracy of DTA by stacking varying numbers of GCN layers.

#### 3.5.1 Analysis of different modules

The difference between the ablation experiments performed in this section lies in the non-identical feature extraction methods. Our baseline approach is to use the Drug Feature Extraction Block for drug molecules and the same CNN Block for protein sequences as in the PFE Block. The CNN Block contains a 1D convolutional layer, a batch normalization layer, and a ReLU layer. In the protein feature fusion stage, the final protein feature vector is obtained simply through direct concatenation. Model-1 utilizes the PFE Block to derive protein features but replaces the original PFF Block with a direct concatenation of the protein’s multi-scale features. Conversely, Model-2 extracts protein sequence features exclusively through the CNN Block and applies the PFF Block to integrate the multi-scale features. The results are shown in Table 5.

**TABLE 5:** Ablation experiments to test the performance of PFF and PFE Blocks.

Dateset	Model	PFE	PFF	MSE ↓	CI ↑	Pearson ↑
Davis	Baseline	-	-	0.228	0.895	0.848
	Model-1	✓	-	0.223	0.895	0.850
	Model-2	-	✓	0.212	0.897	0.859
	MDNN-DTA	✓	✓	0.209	0.901	0.860
KIBA	Baseline	-	-	0.148	0.891	0.885
	Model-1	✓	-	0.146	0.890	0.886
	Model-2	-	✓	0.141	0.892	0.890
	MDNN-DTA	✓	✓	0.135	0.893	0.896

Table 5 shows that our PFE Block (Model-1) improves the performance of the baseline method. The reason is that the proposed module extracts more comprehensive features (both local and global) of the protein, which is essential for accurate and reliable DTA prediction. The experimental results of Model-2 demonstrate that our PFF Block significantly outperforms the simple concatenation approach. This is because we introduced an attention-based weighting parameter, which dynamically adjusts the importance of different features, allowing for a more flexible and comprehensive integration of multi-scale protein features. As a result, the feature fusion module can better integrate the multi-scale features of proteins. It is evident that the independent use of the PFE and PFF blocks yields results not better than those obtained by their combined use. Although both the PFE and PFF blocks provide certain advantages over the baseline model, the results from Model-1 indicate that focusing solely on multi-scale feature extraction without considering the effectiveness of fusion does not lead to high prediction accuracy. Similarly, the results from Model-2 indicate that merely considering the weighted fusion of multi-scale features without emphasizing the completeness of feature extraction also fails to yield optimal results for the model. By combining these two blocks, we find that MDNN-DTA achieves the best performance in the experiments. Compared to the baseline method, the MSE decreases by 8.3 % , and the CI and the Pearson coefficient improve by 0.7 % and 1.4 % , respectively, on the Davis dataset. On the KIBA dataset, the MSE decreases by 8.7 % , and the CI and Pearson coefficient also improve by 0.2 % and 1.2 % , respectively. The integration of these components leads to superior performance on both datasets compared to the baseline method and other configurations.

#### 3.5.2 Component analysis of the protein feature extraction block

The PFE Block contains three components: the Affine Block, the L-R Layer, and the SE-Block. The Affine Block and the L-R Layer are responsible for global feature extraction, while the SE-Block is the attention strategy in the local feature extractor. To understand the role of these components on the performance of the affinity prediction of the model, we conduct detailed ablation experiments on each component in Table 6: 1. Using three convolutional blocks instead of all three components for feature extraction (Model-1); 2. Adding only the SE-Block to Model 1 (Model-2); 3. Adding the Affine block to Model 2 (Model-3).

**TABLE 6:** Comparison of the effects of the components in the PFE Block.

Dateset	Model	Affine	L-R	SE	MSE ↓	CI ↑	Pearson ↑
Davis	Model-1	-	-	-	0.213	0.895	0.845
	Model-2	-	-	✓	0.210	0.897	0.859
	Model-3	✓	-	✓	0.216	0.893	0.845
	MDNN-DTA	✓	✓	✓	0.209	0.901	0.860
KIBA	Model-1	-	-	-	0.141	0.887	0.890
	Model-2	-	-	✓	0.139	0.889	0.892
	Model-3	✓	-	✓	0.143	0.884	0.888
	MDNN-DTA	✓	✓	✓	0.135	0.893	0.896

Comparing the results of Model-1 with Model-2, we can see that the presence of the SE Block improves the result in all three metrics. This is because the SE attention mechanism enhances the representation of features by learning the significance of each channel. It amplifies channels rich in information while suppressing those with less, leading to a more accurate characterization of features. Taking into account the results of Model-2 and Model-3, simply adding the Affine block to the Model-2 configuration does not improve the accuracy of the prediction. Instead, it leads to an increase of 2.7 % in MSE, while CI and Pearson coefficient decrease by 0.4 % and 1.6 % , respectively. The reason for this situation is that the Affine block only applies a linear transformation to the input protein feature vector. Without the support of the L-R layer, it cannot form a MLP to extract protein features according to the amino acid sequence. Comparing the results of Model-2 and Model-3 with MDNN-DTA, we can see that the best experimental results are achieved by the combined application of the Affine Block and L-R Layer (MDNN-DTA). Compared to the single use of Affine blocks for global features extraction, the MSE decreases by 3.2 % , and the CI and the Pearson coefficient improve by 0.83 % and 1.78 % , respectively, on the Davis dataset. On the KIBA dataset, the MSE decreases by 5.5 % , and the CI and the Pearson coefficient also improve by 1.0 % and 0.9 % , respectively. This suggests that Affine blocks do not directly extract global features on their own, but they can rescale the features, and when used in conjunction with a linear layer, can effectively tune and optimize the feature representation, thus allowing the model to capture global information about the protein sequence.

#### 3.5.3 Impact of pre-trained models on prediction results

To validate the effectiveness of the ESM pre-trained model in protein feature extraction, this study design an ablation experiment comparing the model’s prediction performance with and without the ESM-1v pre-trained model. Additionally, we compare ESM-1v with another pre-trained model, ProtT5, to evaluate their differences in prediction accuracy. All experiments are conducted on the Davis dataset, and the results are shown in Table 7. The experimental results show that the ESM-1v pre-trained model significantly improves protein feature extraction. Compared to ProtT5 and the model without pre-training, it better captures the sequence features of proteins and enhances the model’s prediction accuracy. The MSE decreases by 0.04, while the CI value increases by 0.06. ProtT5, as another pre-trained model, performs better than the model without pre-training but still falls short compared to ESM-1v. This may be due to the larger training dataset and more optimized parameters of ESM-1v. The prediction performance of MDNN-DTA without a pre-trained model deteriorates, with the MSE increasing by 0.07 and the CI decreasing by 0.04. This indicates that directly extracting features based on protein sequences may not be sufficient to capture the complex information within the sequences, while the biochemical information in pre-trained models meets the model’s needs and provides more accurate predictions.

**TABLE 7:** Impact of pre-trained models on prediction results.

Dataset	Model	MSE ↓	CI ↑
Davis	Baseline (MDNN-DTA)	0.209	0.901
	Without pre-trained model	0.216	0.897
	With ProtT5	0.213	0.895
	Unweighted fusion	0.211	0.897

**FIGURE 6:**
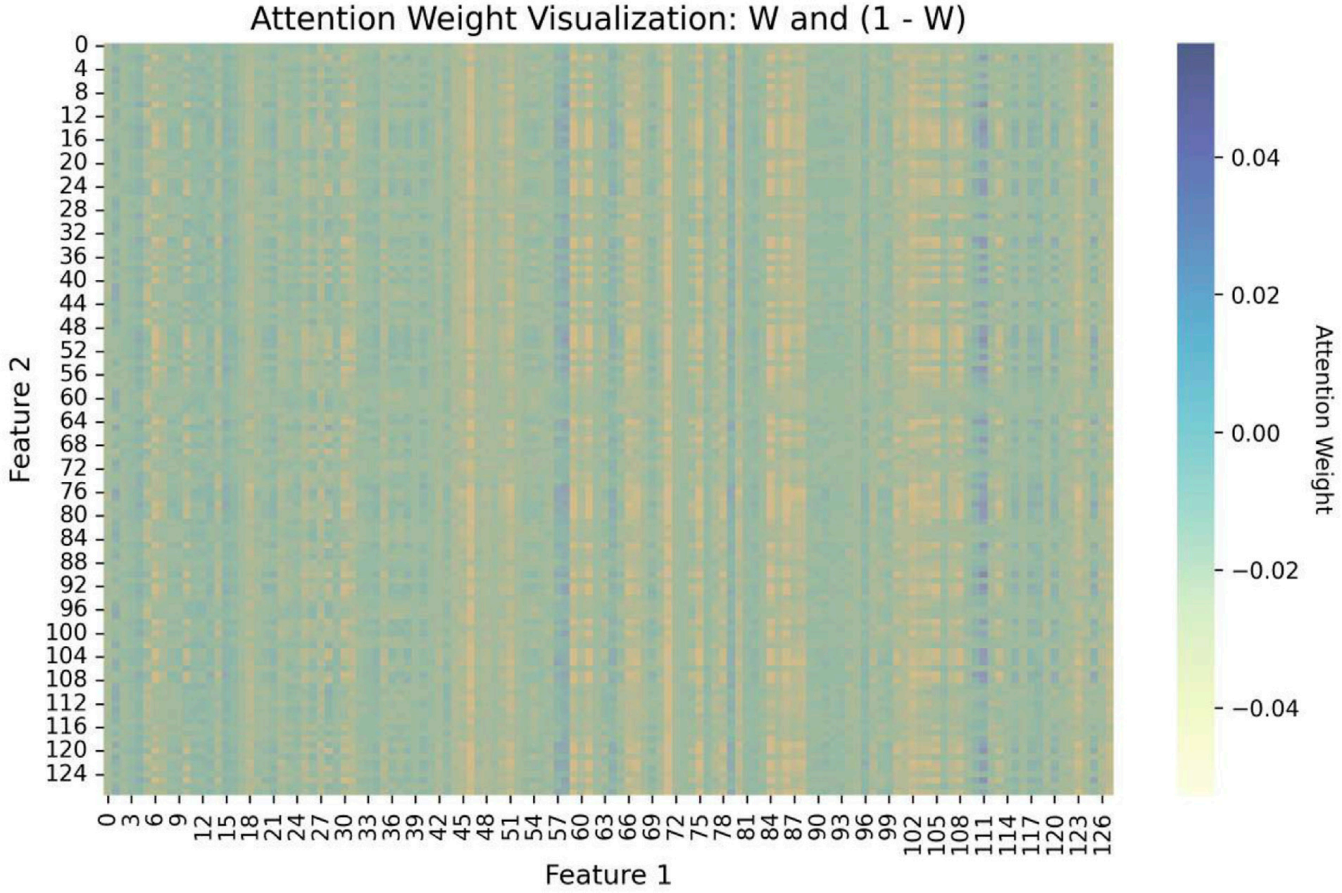
Attention visualization of PFF Block. The blue areas represent higher weights, while the yellow areas represent lower weights.

In MDNN-DTA, we design a PFF block based on the self-attention mechanism to perform weighted fusion of multi-scale features from proteins. To better understand the relationship between different features, we not only visualize the attention weights of the self-attention mechanism but also compare the results with those from the unweighted fusion approach in Table 7. The results show that, without weighted fusion, the model’s prediction accuracy decreases, with the MSE increasing by 0.02 compared to the baseline. Figure 6 illustrates the attention weights ( W and 1−W ) computed during the feature weighted fusion process. From the figure, it can be seen that the model does not distribute attention uniformly across the features; some feature pairs are assigned higher weights, indicating that these feature interactions are more influential in the prediction. The blue areas represent higher weights (stronger attention), while the yellow areas represent lower weights (weaker attention). This weight distribution also indicates that the model dynamically adjusts its attention based on the importance of different features.

### 3.6 Other analysis of the model

#### 3.6.1 Analysis of the number of model parameters and training time

The model parameter size is an important part of deep learning model analysis. While a larger parameter size may improve the model’s accuracy, it also increases training time. To better analyze the effects of parameter size across different models, we design the following models for comparison testing. Baseline-1: The drug sequence uses GCN, while the protein sequence uses CNN for feature extraction. Baseline-2: On top of Baseline-1, the protein branch’s CNN is replaced with Transformer. Model-1: The MDNN-DTA model, using the original architecture. Model-2: The parameter size of the MDNN-DTA original architecture is simplified to that of Baseline-1. Model-3: The parameter size of the MDNN-DTA original architecture is simplified to that of Baseline-2. Model-4: The parameter size of Baseline-2 is expanded to match that of Model-1. The experimental results are shown in Table 8. Baseline-1 and Baseline-2 provide basic comparison benchmarks. While Baseline-1 is faster in training speed, Baseline-2 performs slightly better in MSE and CI, indicating that the Transformer feature extractor is more effective than the traditional CNN method in processing protein sequences. Model-1 provides the best performance (lowest MSE and highest CI), but its total parameter size is significantly higher, and the inference time is also longer, indicating that the complexity of the architecture indeed improves the model’s performance but also increases the computational cost. By simplifying the parameter size of the MDNN-DTA model, Model-2 and Model-3 reduce the computational load while still maintaining high performance. Compared to the baseline-1/2 methods, Model-4 exhibits lower MSE and higher CI results. However, both computation time and MSE increase compared to Model-1 (MDNN). These results suggest that while increasing the parameter size may partially enhance the model’s learning capacity, a well-designed architecture and effective feature extraction methods are also essential for improving performance.

**TABLE 8:** Results of the number of parameters and training time for different models on the different datasets.

Dataset	Model	Params	Training duration	MSE ↓	CI ↑
Davis	Baseline-1	3.7M	1260 s	0.234	0.889
	Baseline-2	4.5M	1470 s	0.223	0.893
	Model-1	6.9M	2030 s	0.209	0.901
	Model-2	3.7M	1260 s	0.211	0.898
	Model-3	4.5M	1480 s	0.213	0.897
	Model-4	6.9M	2050 s	0.214	0.895
KIBA	Baseline-1	3.7M	4290 s	0.151	0.882
	Baseline-2	4.5M	5220 s	0.147	0.884
	Model-1	6.9M	7980 s	0.135	0.893
	Model-2	3.7M	4290 s	0.137	0.891
	Model-3	4.5M	5210 s	0.138	0.890
	Model-4	6.9M	7970 s	0.141	0.889
BindingDB	Baseline-1	3.7M	1530 s	0.627	0.856
	Baseline-2	4.5M	1860 s	0.589	0.861
	Model-1	6.9M	2850 s	0.512	0.873
	Model-2	3.7M	1550 s	0.519	0.868
	Model-3	4.5M	1870 s	0.523	0.866
	Model-4	6.9M	2850 s	0.535	0.863

#### 3.6.2 The effect of the number of layers in a homogeneous network

**FIGURE 7:**
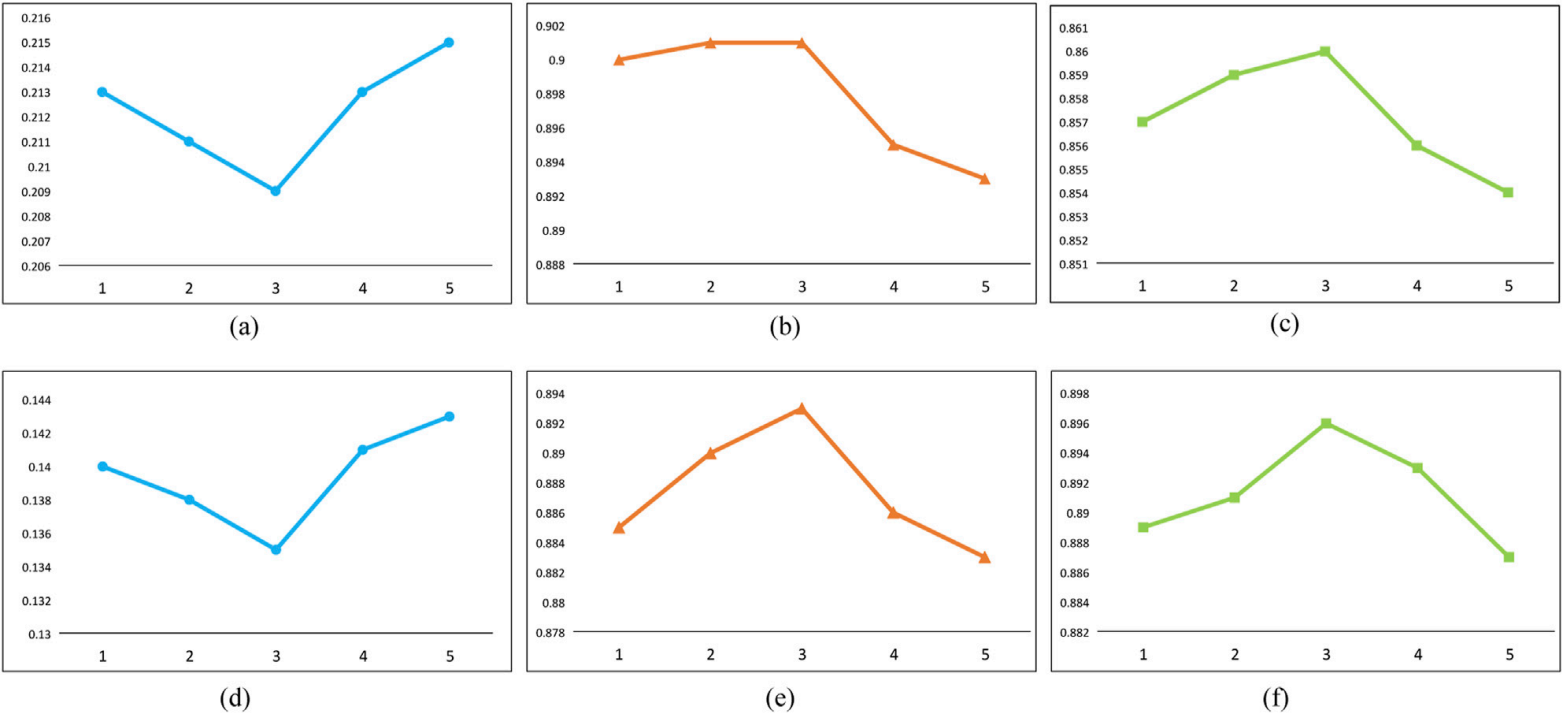
Effect of convolution kernel size on the training process. The horizontal axis represents the training batch, and the vertical axis represents the Loss during the training process. **(A)** MSE results on Davis datasets. **(B)** CI results on Davis datasets. **(C)** Pearson results on Davis datasets. **(D)** MSE results on KIBA datasets. **(E)** CI results on KIBA datasets. **(F)** Pearson results on KIBA datasets.

The precision of drug feature extraction depends on the graph computational capability of the GCN. To evaluate the effect of GCN depth on prediction performance, ablation experiments were conducted, too. Figure 7 demonstrates that a continuous improvement is observed as the number of layers increases to three: the MSE decreases, while both the CI and the Pearson correlation coefficient increase. Conversely, further increasing the number of GCN layers beyond three layers leads to a deterioration in experimental outcomes, yielding results that are inferior to those obtained with a single GCN layer. This trend arises from the fact that each GCN layer primarily processes first-order neighborhood information through spectral graph convolution. While stacking multiple convolutions enables the propagation of higher-order neighborhood information, an excessive number of GCN layers can cause a gradual convergence of node feature vectors within clusters, leading to homogeneity—a phenomenon known as “oversmoothing”. This oversmoothing can negatively impact the model’s decision-making capabilities. Therefore, optimizing the depth of the GCN enhances the efficiency of drug feature extraction, ultimately improving the predictive accuracy of the Multimodal Neural Network for Drug-Target Affinity (MDNN-DTA).

## 4 Conclusion

Accurate DTA prediction can significantly accelerate the process of identifying promising drug candidates in the drug discovery campaign. In this work, we proposed a multimodal deep neural network model for DTA prediction, using different feature extraction blocks to extract effective features from the sequences of drug molecules and target proteins directly. A uniquely designed PFE Block can concurrently capture both global and local features within the protein, while an attention-based PFF Block can integrating multi-scale features of proteins effectively. The superiority of our MDNN-DTA model was demonstrated by comparing with a series of other models. Additionally, ablation experiments gained deeper understanding of the design principles and feasibility of the model components. Altogether, our model shows promising utilization in DTA prediction and provide inspiration to construct new DTA prediction models. Although MDNN-DTA is dedicated to extracting features from the 1D biological sequences of drugs and proteins and has achieved promising results, the contribution of the 3D structure of proteins to affinity prediction cannot be overlooked. We will continue to explore how to integrate the biological 3D structure with the 1D sequences to achieve more accurate affinity prediction results.

## Data Availability

The data presented in the study are deposited in the https://github.com/nuyoaHymf/MDNN-DTA.
