# Supplementary figures and images for: MDNN-DTA: a multimodal deep neural network for drug-target affinity prediction
Source: Front Genet. 2025 Mar 20;16:1527300. doi: 10.3389/fgene.2025.1527300 (PMC11965683; doi:10.3389/fgene.2025.1527300)

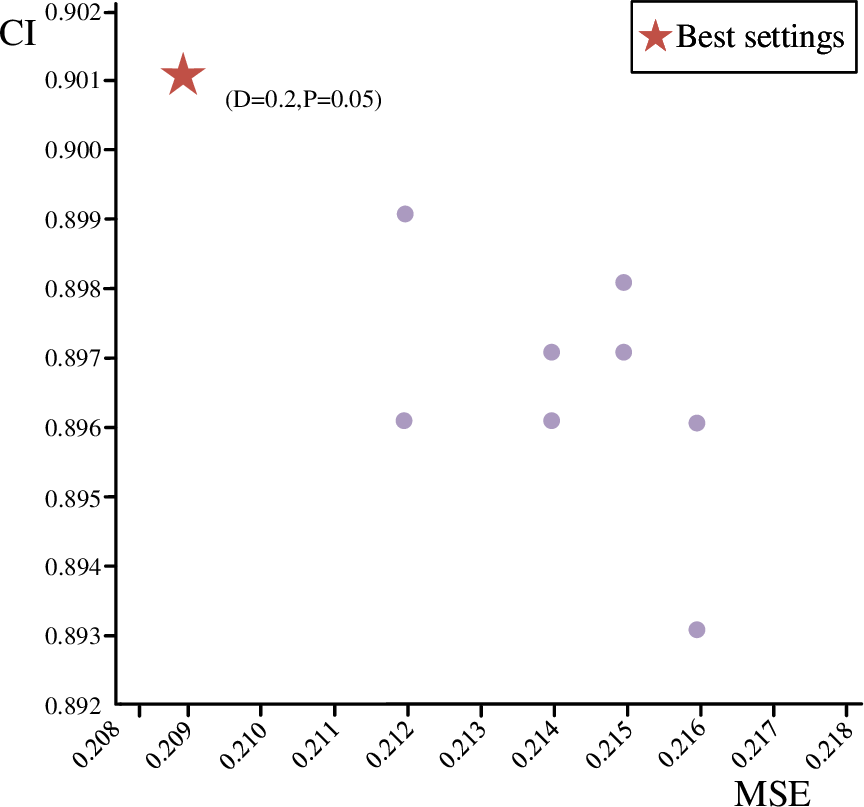

Supplement: Supplementary file 1 [file Image2.tif]

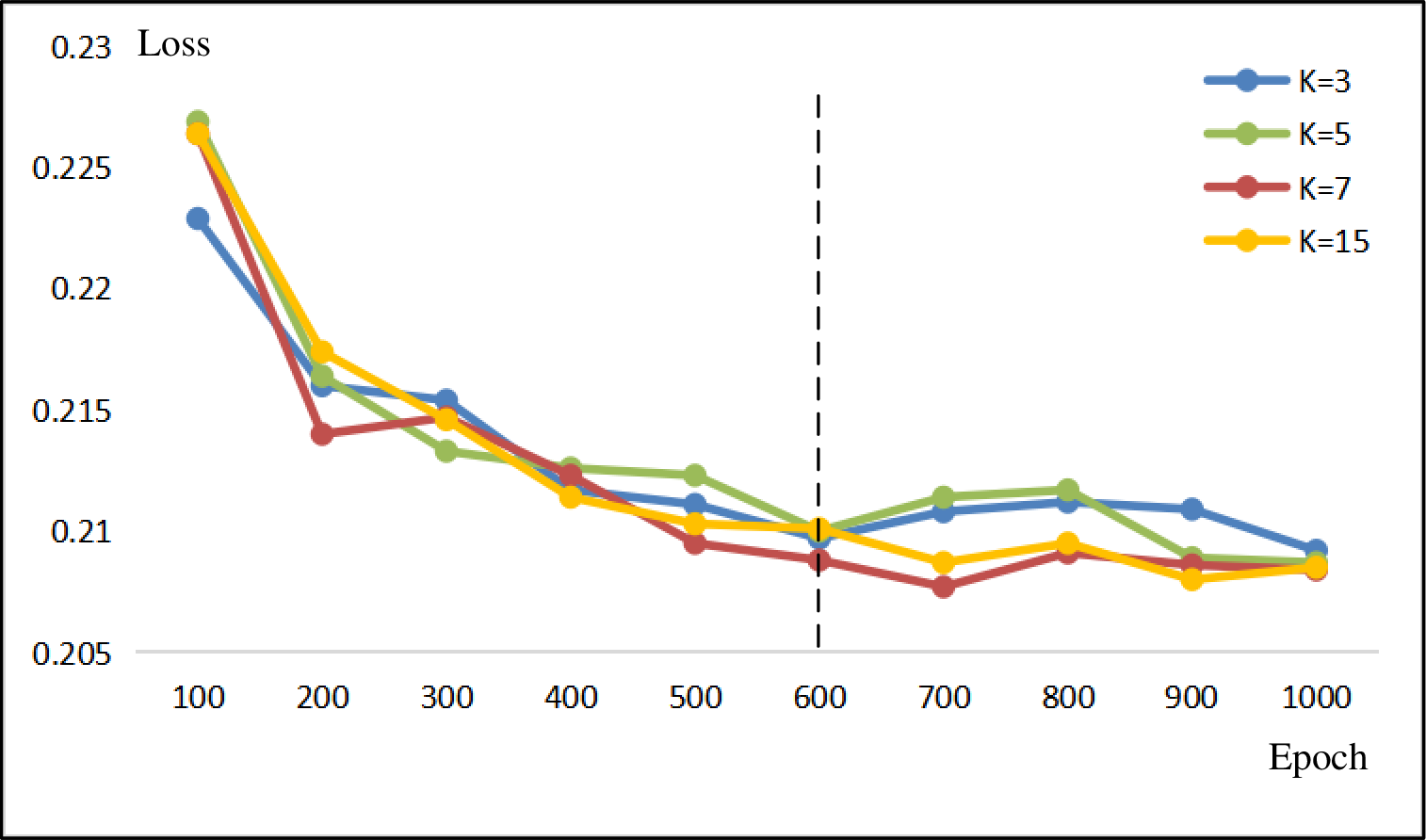

Supplement: Supplementary file 2 [file Image1.tif]
