# Supplementary material for: MDNN-DTA: a multimodal deep neural network for drug-target affinity prediction
Source: Front Genet. 2025 Mar 20;16:1527300. doi: 10.3389/fgene.2025.1527300 (PMC11965683; doi:10.3389/fgene.2025.1527300)
Supplement: Supplementary file 3 [file DataSheet1.pdf]

Supplementary Material

1 SUPPLEMENTARY DATA

In this section, we explore the optimization of two hyperparameters in the MDNN-DTA model. The specific research content is as follows.

1.1 Convolutional kernel size effects in localized feature extractors

The CNN Block is a crucial component of the PFE Block, as it effectively extracts the local features of protein sequences, a capability that primarily relies on the convolutional kernels of the convolutional network. Therefore, we investigate how adjusting the size of the convolutional kernel can improve the model's prediction accuracy. We select 10 nodes from the results of 1000 iterations for experimental demonstration, with all experiments conducted on the Davis dataset. Ideally, the training data continuously fits towards the true values, resulting in the recorded loss values in our log showing an overall decreasing trend. To ensure more accurate analysis, the ten loss values obtained under each parameter setting are averaged with the results of the 20 neighboring experiments. The experimental results are shown in Figure S1.

The four curves in Figure S1 represent the training loss results using convolution kernels of different sizes, with all four experiments starting to converge at epoch 600. The loss curves for the smaller convolution kernels (blue and green) are overall higher than those for the larger convolution kernels (red and yellow), indicating that using larger convolution kernels in the PFE leads to smaller prediction errors. This situation arises because smaller convolution kernels, having a smaller receptive field, focus more on the local amino acid information within the sequence. However, larger convolution kernels, while expanding the receptive field to extract relatively broad contextual information, lose the ability to capture local sequence features. This is particularly evident when comparing the predictions at $K = 7$ and $K = 15$, where the largest convolution kernel demonstrates lower prediction accuracy than the slightly smaller kernel. This phenomenon is related to the protein sequence features that the receptive field of the convolution kernel can cover.

1.2 Impact of dropout probabilities on prediction accuracy

Before executing the DTA prediction phase, it is necessary to concatenate the drug feature vector with the protein feature vector to obtain the final vector representation. To reduce overfitting, dropout is applied after each feature vector is batched. During each forward propagation, a portion of the neurons in the network is ”turned off” with a certain probability P, meaning their outputs do not participate in the computations of subsequent layers. The experimental results using different parameter configurations on the Davis dataset are shown in Table S1, where dropout(P) and dropout(D) represent the dropout values for the protein branch and the drug branch, respectively. For ease of analysis, the experimental results are displayed in a coordinate system, as illustrated in Figure S2.

The results from Table S1 and Figure S2 indicate that the optimal combination of dropout(D) and dropout(P) should be set to 0.2 and 0.05, respectively. This setting achieves the lowest MSE value and the highest CI value, which supports this conclusion. A high dropout rate may cause the loss of important information from drug and protein features due to the random omission of too many neurons, while a low dropout rate can lead to model overfitting. Therefore, this experiment aims to investigate the optimal

combination of the two dropout values that maximizes model accuracy, achieving a balance between preventing overfitting and maintaining learning capacity.

2 SUPPLEMENTARY TABLES AND FIGURES

2.1 Figures

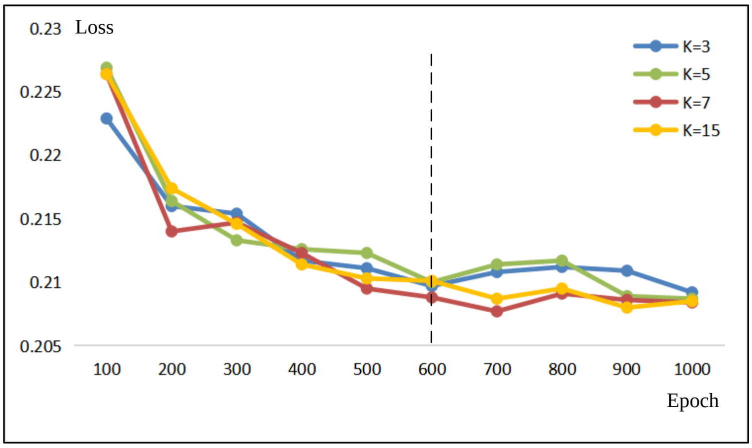

Figure S1. Effect of convolution kernel size on the training process. The horizontal axis represents the training batch, and the vertical axis represents the Loss during the training process.

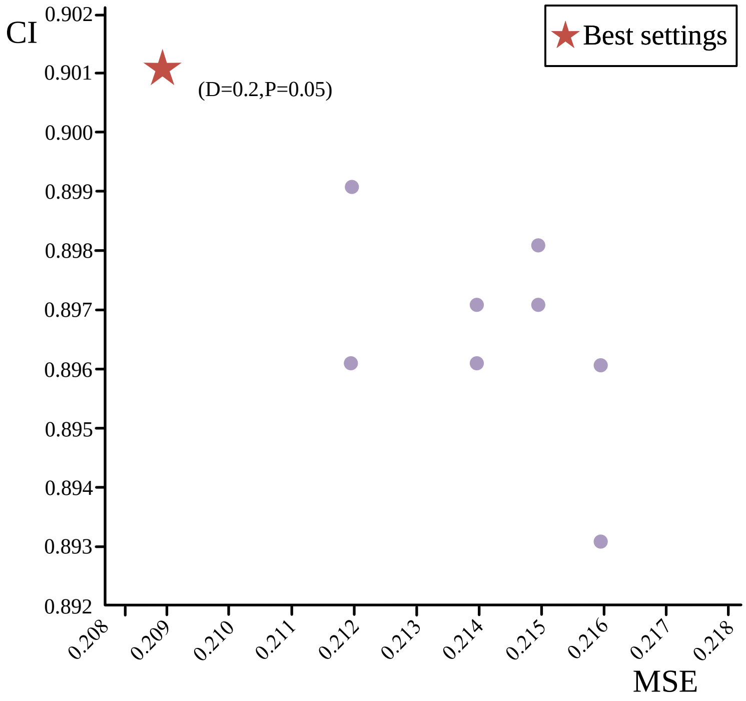

Figure S2. MSE and CI results with different settings. The red pentagram symbol indicates the best setting with a dropout(D) of 0.2 and a dropout(P) of 0.05.

2.2 Tables

Table S1. The results of using different dropout rates for drug and target protein features.

	Dropout(P)=0.05		Dropout(P)=0.1		Dropout(P)=0.2	
	**MSE**	**CI**	**MSE**	**CI**	**MSE**	**CI**
**Dropout(D)=0.05**	0.214	0.897	0.212	0.899	0.215	0.898
**Dropout(D)=0.1**	0.214	0.896	0.215	0.897	0.216	0.896
**Dropout(D)=0.2**	0.209	0.901	0.212	0.896	0.216	0.893
